# Cholecystectomy Increases the Risk of Chronic Kidney Disease: A Nationwide Longitudinal Cohort Study

**DOI:** 10.3390/jcm13216598

**Published:** 2024-11-02

**Authors:** Ji Hye Heo, Eun Ji Kim, Han Na Jung, Kyung-Do Han, Jun Goo Kang, Seong Jin Lee, Sung-Hee Ihm, Eun Roh

**Affiliations:** 1 Department of Internal Medicine, Hallym University Sacred Heart Hospital, Hallym University College of Medicine, Anyang 14068, Republic of Korea; jihyeheo02@gmail.com (J.H.H.); kimu1177@gmail.com (E.J.K.); wgilrw@naver.com (H.N.J.); kjg0804@empas.com (J.G.K.); leesj@hallym.ac.kr (S.J.L.); ihmsh@hallym.or.kr (S.-H.I.); 2 Department of Statistics and Actuarial Science, Soongsil University, Seoul 06978, Republic of Korea; hkd917@naver.com

**Keywords:** holecystectomy, chronic kidney disease, gall bladder, bile acids, cohort study

## Abstract

**Background/Objectives:** Growing evidence suggests that cholecystectomy is associated with adverse health outcomes, including the development of metabolic diseases. However, data on the association between cholecystectomy and kidney disease are limited. The present study aimed to investigate the association between cholecystectomy and chronic kidney disease (CKD) using a nationwide longitudinal cohort. **Methods:** Participants aged ≥20 years with cholecystectomy between 2010 and 2014 (*n* = 116,748) and age- and sex-matched control participants without cholecystectomy (*n* = 116,748) were analyzed using the Korea National Health Insurance Service data. The adjusted hazard ratios (aHRs) were calculated for incident CKD in the cholecystectomy group compared with the nonoperative controls. **Results:** A total of 233,496 participants were included (mean age, 54.7 ± 12.7 years; 52.6% men). During the mean follow-up period of 4.8 ± 1.7 years, 6450 patients (5.5%) were newly diagnosed with CKD in the cholecystectomy group. Cholecystectomy was an independent risk factor for the development of CKD after adjustment for confounders, including age, sex, income, health behaviors, and comorbidities. The risk of CKD was 21% higher in the cholecystectomy group compared to the non-cholecystectomy group (aHR, 1.21; 95% CI, 1.17–1.26). The increased risk of CKD in the cholecystectomy group was consistently significant when a stratified analysis by age, sex, and presence or absence of comorbidities was conducted. **Conclusions:** Cholecystectomy was independently associated with an increased risk of developing CKD in a nationwide population-based study. Therefore, careful and long-term monitoring of the risk of CKD after cholecystectomy is necessary.

## 1. Introduction

Cholecystectomy, the surgical excision of the gallbladder, ranks among the most commonly undertaken surgeries worldwide, primarily for the treatment of various gallbladder pathologies such as cholecystitis, gallstones, and gallbladder neoplasms. In South Korea, cholecystectomy ranks as the fifth most common surgical intervention and is typically associated with a low incidence of immediate postoperative complications [1]. While short-term outcomes are generally favorable, emerging evidence suggests that cholecystectomy may lead to a range of long-term health issues. These include an increased risk of fractures, depression, and neurological conditions such as Parkinson’s disease [2,3,4].

Beyond these conditions, the removal of the gallbladder eliminates its function in bile storage and concentration, which may subsequently disrupt systemic metabolic homeostasis. The gallbladder plays a critical role in the regulation of bile acid metabolism, which influences various metabolic pathways, including those related to glucose, insulin, lipid, and lipoprotein regulation [5]. The absence of this regulatory function has been implicated in abnormal metabolic consequences, with recent studies linking cholecystectomy to the development of metabolic syndrome [6,7,8], non-alcoholic fatty liver disease [9,10], and an elevated risk of type 2 diabetes [11]. These findings underscore the complex metabolic effects that may arise after cholecystectomy, suggesting a broader impact on long-term health.

Chronic kidney disease (CKD) is a significant public health concern, impacting up to 15% of the global population, with its prevalence expected to increase in the coming years [12]. CKD is associated with not only an increased risk of progression to end-stage renal disease (ESRD) but also a heightened risk of cardiovascular disease, even in the early stages of renal impairment [13]. Given the significant burden of CKD, it is crucial to identify both traditional and emerging risk factors for its development. Established metabolic risk factors such as obesity, hyperglycemia, and dyslipidemia are well-documented contributors to CKD [13,14,15]. Meta-analysis results have shown that metabolic syndrome and its components are associated with an increased risk of CKD, including the development of an estimated glomerular filtration rate (eGFR) < 60 mL/min/1.73 m^2^, as well as microalbuminuria or overt proteinuria [15]. Additionally, recent studies have emphasized alterations in bile acid metabolism and gut microbiota alterations as emerging independent risk factors for renal dysfunction [16,17]. Since cholecystectomy may disrupt metabolic balance, potentially leading to these same metabolic abnormalities, it is plausible that the procedure could influence kidney function and contribute to the onset of CKD.

Despite this potential link, no study to date has explored the relationship between cholecystectomy and the development of CKD. Considering the potential metabolic changes induced by the loss of gallbladder function and their known association with kidney disease, investigating this relationship is of critical importance. Therefore, this study aims to examine the association between cholecystectomy and incident CKD using a population-based nationwide database in the Republic of Korea.

## 2. Materials and Methods

### 2.1. Data Sources

This retrospective cohort study utilized data from the National Health Insurance Service (NHIS) database. Managed by the Korean government, the NHIS is the largest health insurance corporation in South Korea, covering approximately 98% of the population since its establishment in 2000 [18]. The NHIS database contains extensive data on its subscribers (excluding foreign nationals), including variables such as sex, age, residential location, socioeconomic factors (e.g., income), and detailed medical claim information, including disease and procedure codes. The NHIS recommends periodic health checkups for its enrollees: biennially for individuals over 40 years of age and annually for employees over 20. These checkups include comprehensive assessments such as medical, surgical, social, and family histories, physical examinations, hearing and vision tests, as well as laboratory investigations.

### 2.2. Study Population

**Figure 1:**
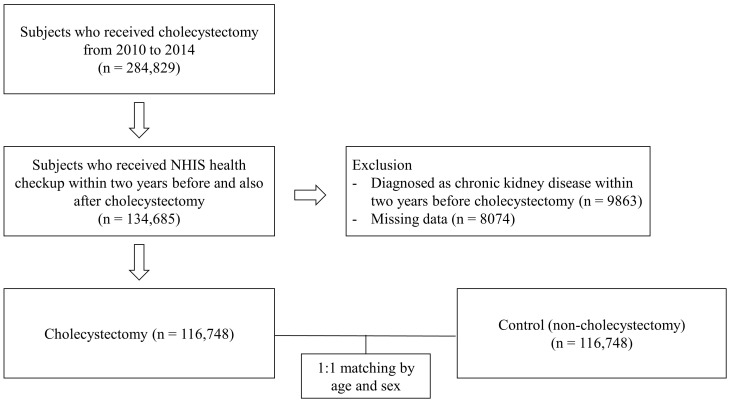
Flow chart of participant enrollment. NHIS, National Health Insurance Service.

Participants aged 20 years or older who underwent cholecystectomy between 2010 and 2014 were selected from the NHIS database, identified using the national health insurance procedure code for cholecystectomy (Q7380) [9]. We further narrowed the selection to individuals who had undergone NHIS health checkups both within two years prior to and following cholecystectomy, resulting in a total of 134,685 participants. After excluding those with a pre-existing diagnosis of CKD (*n* = 9863) and individuals with missing data (*n* = 8074), 116,748 participants remained. A control group of 116,748 individuals, matched 1:1 by age and sex, was recruited from the NHIS database among those who did not undergo cholecystectomy. For participants who had cholecystectomy, the index date was defined as the date of the procedure, and the same index date was assigned to each matched control. This yielded a final cohort of 233,496 participants (116,748 cases and 116,748 controls). A flow chart illustrating the participant selection process is shown in Figure 1. The participants were followed until either the onset of CKD or December 31, 2019, whichever occurred first.

The study protocol was approved by the Institutional Review Board of Hallym University College of Medicine (IRB No. HALLYM 2021-06-026) and was conducted in accordance with the principles of the Declaration of Helsinki. The requirement for informed consent was waived due to the use of anonymized and de-identified data in accordance with the confidential guidelines of the NHIS of Korea.

### 2.3. Data Collection

Detailed demographic and lifestyle information was collected from the participants through standardized self-reported questionnaires. Low income was defined as being in the lowest 25% of the income distribution. Smoking status was categorized as non-smoker, ex-smoker, or current smoker. Alcohol consumption was classified into three groups: none (0 g/day), mild (<30 g/day), and heavy (>30 g/day). Regular exercise was defined as engaging in high-intensity physical activity at least three times per week or moderate-intensity physical activity at least five times per week. The NHIS health checkup included both physical examinations and laboratory tests. Height, weight, and waist circumference (WC) were measured, and body mass index (BMI) was calculated by dividing weight by the square of height (kg/m^2^). Systolic and diastolic blood pressures (BPs) were recorded with participants seated and after at least five minutes of rest. Fasting blood samples were analyzed to assess glucose, total cholesterol, triglyceride (TG), high-density lipoprotein cholesterol (HDL-C), low-density lipoprotein cholesterol (LDL-C), and creatinine levels using enzymatic methods.

### 2.4. Study Outcomes and Definition of Comorbidities

The primary outcome of this study was the incidence of newly developed chronic kidney disease (CKD) during follow-up. CKD was defined as an eGFR < 60 mL/min/1.73 m^2^ [19]. The eGFR was calculated using the CKD Epidemiology Collaboration (CKD-EPI) equation [20].

Hypertension was defined as a blood pressure (BP) ≥140/90 mmHg or at least one annual claim for a prescription of antihypertensive medication, based on ICD-10 codes I10–I15 [9]. Dyslipidemia was defined as a total cholesterol level ≥240 mg/dL or at least one annual claim for a prescription of lipid-lowering medication under ICD-10 code E78 [21]. Diabetes was defined as a fasting blood glucose level ≥126 mg/dL or a prescription for antidiabetic medication, according to ICD-10 codes E11–E14 [22]. Obesity was defined as a body mass index (BMI) ≥25 kg/m^2^, following the Asia-Pacific criteria outlined by the World Health Organization [23].

### 2.5. Statistical Analyses

The baseline characteristics of the participants are presented as means ± standard deviations or medians (interquartile ranges) for continuous variables and as numbers (percentages) for categorical variables. Independent *t*-tests were used to compare continuous variables between the two groups, while chi-square tests were employed for comparisons of categorical variables. We assessed the association between cholecystectomy and the incidence of new CKD cases during follow-up. Multivariable-adjusted models accounted for potential confounders, including age, sex, low income, smoking status, alcohol consumption, regular exercise, diabetes, hypertension, dyslipidemia, fasting glucose levels, WC, and BMI. The results are reported as hazard ratios (HRs) with 95% confidence intervals (CIs), comparing the cholecystectomy group to the non-cholecystectomy group, which served as the reference. Additionally, the relationship between cholecystectomy and CKD development was examined across various subgroups based on age, sex, smoking status, alcohol consumption, regular exercise, and the presence of baseline dyslipidemia, obesity, diabetes, and hypertension. Statistical significance was defined as *p* < 0.05. All statistical analyses were performed using SAS version 9.4 (SAS Institute Inc., Cary, NC, USA).

## 3. Results

### 3.1. Baseline Characteristics of the Study Population

The study population comprised 233,496 participants, including 116,748 individuals who underwent cholecystectomy between 2010 and 2014 and 116,748 matched controls without cholecystectomy. The baseline characteristics of the participants with and without cholecystectomy are presented in Table 1. The mean age of the cohort was 54.7 ± 12.7 years, with 52.6% of the participants being male. The participants in the cholecystectomy group had higher BMI, WC, fasting glucose, and triglyceride levels but lower HDL-C levels compared to those in the non-cholecystectomy group (all *p* < 0.001). Baseline eGFR, LDL-C, and BP were comparable between the two groups. Additionally, the cholecystectomy group had a higher prevalence of hypertension, dyslipidemia, and diabetes at baseline (all *p* < 0.001). The proportions of non-smokers and regular exercisers were lower in the cholecystectomy group, while the proportion of non-drinkers was higher, compared to the non-cholecystectomy group (all *p* < 0.001).

### 3.2. Risk of Incident CKD According to Cholecystectomy Status

Among the 116,748 patients who underwent cholecystectomy, 6450 (5.5%) developed new-onset CKD during a mean follow-up period of 4.8 ± 1.7 years. The cholecystectomy group exhibited a 29% higher risk of developing CKD compared to the non-cholecystectomy group (unadjusted HR, 1.29; 95% CI, 1.24–1.34) (Table 2). After adjusting for potential confounders, including age, sex, income, smoking status, alcohol consumption, regular exercise, diabetes, hypertension, dyslipidemia, fasting glucose, WC, and BMI, the increased risk of CKD in the cholecystectomy group remained significant (adjusted HR, 1.21; 95% CI, 1.17–1.26).

### 3.3. Risk of Incident CKD According to Cholecystectomy Status in Subgroups

We assessed the risk of incident CKD in the cholecystectomy group through subgroup analyses, stratified by age, sex, smoking status, alcohol consumption, regular exercise, and comorbidities including dyslipidemia, obesity, diabetes, and hypertension (Table 3). A consistently elevated risk of CKD following cholecystectomy was observed across all subgroups. Notably, the increased risk of developing CKD following cholecystectomy was significantly higher in participants without obesity (BMI < 25) compared to those with obesity (BMI ≥ 25) (unadjusted HR, 1.34; 95% CI, 1.27–1.41 in participants without obesity vs. unadjusted HR, 1.16; 95% CI, 1.09–1.23 in participants with obesity, *p* for interaction = 0.001). After adjusting for age, sex, income, smoking status, alcohol consumption, regular exercise, comorbidities, fasting glucose, WC, and BMI, the positive association between cholecystectomy and incident CKD remained consistent across all subgroups. The increased risk of CKD development in the cholecystectomy group was higher in individuals without obesity compared with those with obesity (adjusted HR, 1.33; 95% CI, 1.25–1.39 in participants without obesity vs. adjusted HR, 1.20; 95% CI, 1.14–1.28 in participants with obesity, *p* for interaction = 0.023).

## 4. Discussion

In this large-scale longitudinal cohort study of 233,496 participants from the general Korean population, we explored the association between cholecystectomy and the development of CKD. Our findings indicate that individuals who underwent cholecystectomy had an approximately 21% higher risk of developing CKD compared to those who did not undergo the procedure. Cholecystectomy remained an independent predictor of incident CKD even after adjusting for multiple confounding factors. Notably, the elevated risk of CKD in individuals who had undergone cholecystectomy was consistent across various subgroups based on age, sex, smoking and drinking status, and presence of comorbidities such as obesity, hypertension, diabetes, and dyslipidemia. These results suggest that the absence of the gallbladder may serve as a potential risk factor for the development of CKD. To our knowledge, this is the first and largest nationwide longitudinal cohort study to demonstrate a significant relationship between cholecystectomy and CKD.

Cholecystectomy is the gold standard for the treatment of various gallbladder disorders, particularly symptomatic gallstone diseases. However, recent studies have raised concerns about several potential adverse health outcomes following cholecystectomy [2,3,4,6,8,9,24,25,26]. Notably, growing evidence supports a link between cholecystectomy and the development of metabolic disorders, including non-alcoholic fatty liver disease, metabolic syndrome, and type 2 diabetes [6,8,9,24,25,26]. A prior study reported a significant postoperative weight gain in cholecystectomized patients, with men and women gaining an average of 4.6% and 3.3% of their preoperative body weight, respectively, six months after surgery [27]. In addition, a comparative analysis revealed that individuals with gallstones, particularly those who had undergone cholecystectomy, exhibited higher BMI and waist circumference, with a significantly greater prevalence of metabolic syndrome in the subjects with cholecystectomy (63.5%) compared to those with gallstones (47.0%) or without gallstone disease (30.3%; *p* < 0.01 for both) [28]. Another cross-sectional study demonstrated that patients who underwent cholecystectomy had a significantly higher prevalence of cardiovascular risk factors, including type 2 diabetes, hypertension, and hypercholesterolemia, compared with controls [10]. Moreover, cholecystectomy, but not gallstones, is independently associated with NAFLD [7,8]. However, the majority of prior studies were limited by small sample sizes and cross-sectional designs. Moreover, these studies were unable to examine the isolated effect of cholecystectomy on future metabolic health, as they did not distinguish between the impact of cholecystectomy and that of gallstone disease. A recent review indicated that cholecystectomy itself may induce metabolic abnormalities, making cholecystectomized individuals metabolically distinct from gallstone patients with a retained gallbladder [5]. A recent Korean longitudinal cohort study found that cholecystectomy is an independent risk factor for metabolic syndrome and type 2 diabetes in the general population [6,9]. Given that metabolic abnormalities are key drivers of CKD onset, understanding the relationship between cholecystectomy and CKD is crucial. However, few studies have examined the impact of cholecystectomy on long-term renal outcomes.

Under normal circumstances, the gallbladder plays a critical role in regulating the enterohepatic circulation of bile acids, controlling their flow and release into the intestine, which is essential for maintaining metabolic and physiological homeostasis [29]. Following cholecystectomy, the rhythm and rate at which bile acids enter the intestine are disrupted. Berr et al. reported a 16% reduction in the bile acid pool three months after cholecystectomy [30], while other in vivo studies demonstrated a more substantial decrease of approximately 40% within two weeks post-surgery [31,32]. In addition to the reduction in the bile acid pool, the balance of bile acid metabolism is disturbed due to a decreased expression of fibroblast growth factor (FGF), which affects the intricate interaction between bile acids and the intestinal microbiota [33]. This disruption can activate inflammatory pathways, including NF-kB and interleukin-17, leading to the expression of pro-inflammatory genes [34]. Furthermore, the changes in the gut microbiota following cholecystectomy may activate Toll-like receptors, promoting the production of reactive oxidative species and contributing to oxidative stress [35]. Based on these findings, we hypothesize that the increased risk of CKD after cholecystectomy may be linked to alterations in bile acid metabolism, gut microbiota composition, and the complex interactions between these factors.

The exact mechanisms by which cholecystectomy contributes to the development of CKD remain unclear. Cholecystectomy alters the rhythm and amount of bile acids entering the intestine, contributing to metabolic disturbances [36]. Emerging research has highlighted bile acid metabolism and gut microbiota alterations as potential independent risk factors for renal dysfunction [16,17]. Xiao et al. reported that reduced bile acid levels were independently associated with an increased risk of end-stage renal disease in patients with diabetic kidney disease [16]. Another study found that differences in the gut microbiota related to bile acid metabolism were associated with CKD in patients with hypertension [17]. Supporting these findings, a recent study demonstrated that a farnesoid X receptor agonist, which regulates bile acid homeostasis and cholesterol-derived bile acid biosynthesis, had a beneficial effect on renal outcomes in patients with non-alcoholic steatohepatitis [37]. These studies align with our findings, further substantiating the link between cholecystectomy and CKD. However, further research is necessary to fully elucidate the physiological changes following cholecystectomy and their impact on renal function.

The present study clearly establishes a strong association between cholecystectomy and the incidence of CKD. Individuals who underwent cholecystectomy had poorer baseline metabolic profiles compared to those who did not, which could independently influence future renal function. However, after adjusting for a wide range of metabolic parameters and performing subgroup analyses stratified by the presence or absence of various metabolic risk factors, a consistently higher risk of incident CKD was observed in the cholecystectomy group. These findings suggest that cholecystectomy itself may contribute to an increased risk of CKD, independently of the baseline metabolic characteristics observed in cholecystectomy patients. Notably, the heightened risk of CKD following cholecystectomy was more pronounced in participants without obesity. Since obesity is a well-known risk factor for CKD [13,14,15,38], it is possible that the presence of obesity attenuates the impact of cholecystectomy on CKD development. Alternatively, these findings may be related to the ‘obesity paradox’. A meta-analysis demonstrated a U-shaped relationship between BMI and all-cause mortality, with the lowest mortality observed in individuals with a BMI in the range of 25–30 kg/m^2^ [39]. This phenomenon suggests that overweight or mild obesity may have a protective effect in certain populations. This observation highlights the need for careful monitoring of renal function in individuals with fewer traditional risk factors for CKD after undergoing cholecystectomy. These findings underscore the importance of vigilance in assessing renal outcomes even in populations considered to have a lower baseline risk for CKD.

There are several limitations to the present study. First, due to the observational design, we cannot establish a definitive causal relationship between cholecystectomy and CKD. Additionally, the follow-up period may be insufficient to fully assess the long-term cause–effect relationship between cholecystectomy and incident CKD. Second, we did not account for changes in other clinical parameters, interventions, or medications affecting renal function during the follow-up period, which could influence the results. Moreover, our study lacks data on the use of nephrotoxic agents, such as nonsteroidal anti-inflammatory drugs and radiocontrast media, which may confound the relationship between cholecystectomy and CKD. Additionally, although patients with baseline CKD were excluded to reduce confounding, we did not specifically exclude individuals with a history of renal stones, polycystic kidney disease, or prior renal surgery, due to a lack of available data on these conditions. These conditions may influence renal function and affect the observed relationship between cholecystectomy and CKD. Third, although the short-term postoperative outcomes are generally favorable, and a direct link between postoperative morbidity and CKD is difficult to establish, the possibility of early CKD development cannot be excluded. To mitigate this potential confounding factor, future studies should exclude patients who develop CKD within 1–2 years post-cholecystectomy, ensuring a more accurate assessment of the long-term effects of cholecystectomy on renal function. Fourth, as the majority of the study participants were Korean, the generalizability of the findings to other ethnic groups may be limited. Fifth, we were unable to include the albumin-to-creatinine ratio in defining CKD, as this measure was not routinely performed in NHIS health examinations. This limitation may have led to the inclusion of patients with undiagnosed CKD, particularly those with micro- or macroalbuminuria and a GFR > 60 mL/min/1.73 m^2^. Furthermore, the definition of incident CKD relied on a single eGFR measurement using a serum creatinine-based equation, which may not fully capture kidney function. In addition, the higher prevalence of hypertension, dyslipidemia, and diabetes in the cholecystectomy group could have increased the risk of undiagnosed CKD in this population. Despite these limitations, the primary strength of our study lies in the use of a large, nationally representative cohort from Korea, providing robust evidence of an association between cholecystectomy and the development of CKD in the general population. To our knowledge, this study offers the first evidence suggesting that cholecystectomy may contribute to the risk of CKD.

## 5. Conclusions

This nationwide cohort study demonstrated an association between cholecystectomy and an increased risk of developing CKD in cholecystectomized patients compared to those who did not undergo the surgery. Importantly, the increased risk was observed regardless of baseline CKD risk factors, suggesting a potential link between cholecystectomy and CKD onset. However, unmeasured confounding factors cannot be entirely excluded. Given that cholecystectomy is one of the most commonly performed surgeries worldwide, careful long-term monitoring of renal function is recommended for patients following cholecystectomy. Further prospective research should be performed to validate our findings using other datasets and to investigate the potential role of bile acids and their interactions with the gut microbiota in the pathogenesis of CKD.

## Figures and Tables

**Table 1:** Baseline characteristics of the participants.

	Cholecystectomy	Non-Cholecystectomy	*p* Value
	*n* = 116,748	*n* = 116,748	
Age (years)	54.7 ± 12.7	54.7 ± 12.7	1.000
Men (%)	61,426 (52.6)	61,426 (52.6)	1.000
Body mass index (kg/m^2^)	24.6 ± 3.3	23.9 ± 3.1	<0.001
Waist circumference (cm)	83.1 ± 9.1	81.0 ± 8.9	<0.001
Fasting blood glucose (mg/dL)	101.2 ± 25.6	99.3 ± 23.5	<0.001
Total cholesterol (mg/dL)	195.1 ± 37.7	196.4 ± 36.9	<0.001
Triglyceride (mg/dL)	115.6 (115.3–116.0)	111.9 (111.6–112.3)	<0.001
HDL-C (mg/dL)	53.0 ± 18.0	55.1 ± 17.6	<0.001
LDL-C (mg/dL)	115.8 ± 35.7	115.7 ± 35.2	0.556
eGFR (mL/min/1.73 m^2^)	89.8 ± 25.2	90.0 ± 24.1	0.070
Systolic blood pressure	123.7 ± 14.8	123.4 ± 15.1	0.032
Diastolic blood pressure	76.8 ± 9.9	76.6 ± 10.0	0.359
Comorbidities (%)			
Hypertension	43,631 (37.4)	39,022 (33.4)	<0.001
Dyslipidemia	29,700 (25.4)	27,058 (23.2)	<0.001
Diabetes mellitus	17,152 (14.7)	13,011 (11.1)	<0.001
Smoking status			<0.001
Non	71,492 (61.2)	72,736 (62.3)	
Ex	20,740 (17.8)	19,775 (16.9)	
Current	24,516 (21.0)	24,237 (20.8)	
Alcohol			<0.001
None	69,511 (59.5)	65,826 (56.4)	
Mild	39,382 (33.7)	42,709 (36.6)	
Heavy	7855 (6.7)	8213 (7.0)	
Regular exercise	22,713 (19.5)	23,651 (20.3)	<0.001
Low income (<25%)	21,567 (18.5)	22,808 (19.5)	<0.001

Data are expressed as mean ± SD or median (interquartile range) for continuous variables or as *n* (%) for categorical variables. HDL-C, high-density lipoprotein cholesterol; LDL-C, low-density lipoprotein cholesterol; eGFR, estimated glomerular filtration rate; SD, standard deviation.

**Table 2:** Risk of incident chronic kidney disease according to cholecystectomy status.

Cholecystectomy Status	Event (%)	HR (95% CI)		
		Unadjusted	Model 1	Model 2
Non-cholecystectomy	5068 (4.3)	1 (reference)	1 (reference)	1 (reference)
Cholecystectomy	6450 (5.5)	1.29 (1.24–1.34)	1.30 (1.25–1.35)	1.21 (1.17–1.26)

Model 1: age, sex, low income, smoking status, alcohol intake, and regular exercise. Model 2: age, sex, low income, smoking status, alcohol intake, regular exercise, diabetes, hypertension, dyslipidemia, fasting glucose, waist circumference, and body mass index. CI, confidence interval; HR, hazard ratio.

**Table 3:** Odds ratios and 95% confidence intervals for incident chronic kidney disease according to cholecystectomy status in subgroups.

Subgroup		*n*	Event (%)	Unadjusted HR (95% CI)	*p* for Interaction	^a^ Adjusted HR (95% CI)	*p* for Interaction
Age					0.643		0.790
<65 years	Non-cholecystectomy	88,556	2246 (2.5)	1 (Reference)		1 (Reference)	
	Cholecystectomy	88,556	2920 (3.3)	1.31 (1.24–1.39)		1.22 (1.15–1.29)	
≥65 years	Non-cholecystectomy	28,192	2822 (10.0)	1 (Reference)		1 (Reference)	
	Cholecystectomy	28,192	3530 (12.5)	1.29 (1.22–1.36)		1.21 (1.14–1.27)	
Sex					0.388		0.291
Men	Non-cholecystectomy	61,426	2586 (4.2)	1 (Reference)		1 (Reference)	
	Cholecystectomy	61,426	3242 (5.3)	1.27 (1.2–1.34)		1.19 (1.12–1.25)	
Women	Non-cholecystectomy	55,322	2482 (4.5)	1 (Reference)		1 (Reference)	
	Cholecystectomy	55,322	3208 (5.8)	1.31 (1.24–1.38)		1.24 (1.17–1.31)	
Current smoker					0.574		0.644
No	Non-cholecystectomy	92,511	4255 (4.6)	1 (Reference)		1 (Reference)	
	Cholecystectomy	92,232	5424 (5.9)	1.3 (1.24–1.35)		1.22 (1.17–1.27)	
Yes	Non-cholecystectomy	24,237	813 (3.4)	1 (Reference)		1 (Reference)	
	Cholecystectomy	24,516	1026 (4.2)	1.26 (1.15–1.38)		1.19 (1.08–1.31)	
Regular drinker					0.823		0.316
None	Non-cholecystectomy	65,826	3508 (5.3)	1 (Reference)		1 (Reference)	
	Cholecystectomy	69,511	4642 (6.7)	1.27 (1.22–1.33)		1.23 (1.17–1.29)	
Yes	Non-cholecystectomy	50,922	1560 (3.1)	1 (Reference)		1 (Reference)	
	Cholecystectomy	47,237	1808 (3.8)	1.26 (1.18–1.35)		1.18 (1.10–1.26)	
Regular exercise					0.495		0.064
No	Non-cholecystectomy	93,097	4031 (4.3)	1 (Reference)		1 (Reference)	
	Cholecystectomy	94,035	5151 (5.5)	1.28 (1.23–1.34)		1.21 (1.16–1.26)	
Yes	Non-cholecystectomy	23,651	1037 (4.4)	1 (Reference)		1 (Reference)	
	Cholecystectomy	22,713	1299 (5.7)	1.32 (1.22–1.44)		1.23 (1.13–1.34)	
Dyslipidemia					0.087		0.063
No	Non-cholecystectomy	89,690	3309 (3.7)	1 (Reference)		1 (Reference)	
	Cholecystectomy	87,048	3942 (4.5)	1.24 (1.18–1.3)		1.18 (1.12–1.24)	
Yes	Non-cholecystectomy	27,058	1759 (6.5)	1 (Reference)		1 (Reference)	
	Cholecystectomy	29,700	2508 (8.4)	1.33 (1.25–1.41)		1.27 (1.19–1.36)	
Obesity					0.001		0.023
No	Non-cholecystectomy	77,315	2949 (3.8)	1 (Reference)		1 (Reference)	
	Cholecystectomy	66,361	3342 (5.0)	1.34 (1.27–1.41)		1.33 (1.25–1.39)	
Yes	Non-cholecystectomy	39,433	2119 (5.4)	1 (Reference)		1 (Reference)	
	Cholecystectomy	50,387	3108 (6.2)	1.16 (1.09–1.23)		1.20 (1.14–1.28)	
Diabetes					0.682		0.646
No	Non-cholecystectomy	103,737	3835 (3.7)	1 (Reference)		1 (Reference)	
	Cholecystectomy	99,596	4515 (4.5)	1.24 (1.18–1.29)		1.22 (1.16–1.27)	
Yes	Non-cholecystectomy	13,011	1233 (9.5)	1 (Reference)		1 (Reference)	
	Cholecystectomy	17,152	1935 (11.3)	1.22 (1.13–1.31)		1.19 (1.10–1.29)	
Hypertension					0.813		0.952
No	Non-cholecystectomy	77,726	1962 (2.5)	1 (Reference)		1 (Reference)	
	Cholecystectomy	73,117	2270 (3.1)	1.24 (1.16–1.32)		1.21 (1.14–1.29)	
Yes	Non-cholecystectomy	39,022	3106 (8.0)	1 (Reference)		1 (Reference)	
	Cholecystectomy	43,631	4180 (9.6)	1.23 (1.17–1.29)		1.21 (1.15–1.27)	

^a^ Adjusted by age, sex, low income, smoking status, alcohol intake, regular exercise, diabetes, hypertension, dyslipidemia, fasting glucose, waist circumference, and body mass index.

## Data Availability

The datasets generated and/or analyzed during the current study are available in the National Health Insurance Sharing Service repository, [https://nhiss.nhis.or.kr/bd/ay/bdaya001iv.do, accessed on 28 September 2024].
